# Inconsistent Findings Between Crystal Violet and Congo Red Methods on Biofilms with Comparative Sugar Supplementation

**DOI:** 10.3390/microorganisms14010021

**Published:** 2025-12-21

**Authors:** Nihan Unubol, Meltem Ayaş, Neval Yurttutan Uyar, Erkan Mozioğlu

**Affiliations:** 1 Department of Medical Laboratory Techniques, Vocational School of Health Services, Acibadem Mehmet Ali Aydinlar University, Istanbul 34752, Türkiye; 2 Department of Medical Microbiology, School of Medicine, Acibadem Mehmet Ali Aydinlar University, Istanbul 34752, Türkiye; 3 Acibadem Labmed Medical Laboratories, Istanbul 34752, Türkiye; 4 Department of Medical Biotechnology, Graduate School of Health Sciences, Acibadem Mehmet Ali Aydinlar University, Istanbul 34752, Türkiye

**Keywords:** biofilm, Congo Red, crystal violet, comparison, inconsistent

## Abstract

In recent years, the World Health Organization has highlighted biofilm-derived multidrug-resistant bacteria as a critical threat to both global health and the environment. Although various testing methods are available, crystal violet and Congo Red methods are among the most frequently used methods for biofilm detection in the literature. However, inconsistent findings across studies have raised concerns. To address these issues and offer valuable insights for researchers in the field, this study used clinically relevant standard bacterial strains (ATCC or NCTC strains) to perform biofilm assays with both methods and compare the results. To investigate the effect of different sugar sources on biofilm formation, various sugar substrates were also examined using both biofilm methods under controlled culture conditions in this study. When the results were evaluated, significant differences were found between the two methods closely related to sugar content. Of the 22 strains tested, 17 (77%) showed different biofilm results in a sugar-free environment. Similar inconsistencies were also observed with glucose (32% of strains) and sucrose (50% of strains). With fructose, some strains (*P. aeruginosa* strains, *E. faecalis* ATCC 29212, *K. pneumoniae* High Level ESBL, *K. pneumoniae* BAA 1706, *A. baumannii* BAA 747) were negative with Congo Red and positive with crystal violet, while others (*S. mutans* ATCC 25175, *E. coli* NCTC 13846, *E. coli* ATCC 25922) gave the opposite results. Fructose caused inconsistencies in approximately 45% of strains. The highest agreement between the two methods (approximately 68%) was observed when glucose was used as the carbon source.

## 1. Introduction

In recent years, the World Health Organization has recognized biofilm-derived multidrug-resistant bacteria as a major threat to global health and the environment [1,2,3]. A lack of awareness within the medical field regarding the frequent presence of bacteria in biofilm form and the significant clinical implications of this—has increasingly affected global public health and contributed to the rise in chronic diseases. Understanding the role of biofilms in infections is essential for healthcare professionals to manage these conditions appropriately and effectively [4]. Therefore, it is critical to monitor bacterial biofilms and develop new strategies to combat biofilm-associated infections. Recent research highlights the importance of real-time monitoring of biofilm formation [5].

Biofilm refers to a structured community of microbial cells that adhere to living or non-living surfaces and are embedded within an extracellular polymeric substance (EPS) [6]. Biofilm formation is a cyclical process involving four main stages: adhesion, colonization, maturation, and disintegration [7]. Biofilms pose a significant threat to public health due to their extreme resistance to the human immune system and high concentrations of antibiotics, as well as their ability to neutralize these agents and cause persistent, destructive tissue inflammation [4,8]. According to recent studies, the extracellular matrix in biofilms impedes the migration of leukocytes through the biofilm, thereby inhibiting the production of reactive oxygen species (ROS) that are essential for bacterial phagocytosis [9,10,11]. Research has shown that clinically relevant bacterial pathogens such as *Enterobacter cloacae*, *Escherichia coli*, *Klebsiella pneumoniae*, *Pseudomonas aeruginosa*, *Staphylococcus aureus*, and *S. epidermidis* exhibit a high capacity for biofilm formation [7]. These bacteria frequently colonize medical devices such as catheters, prostheses, and mechanical heart valves, leading to persistent infections and potentially permanent damage. Moreover, biofilms contribute to bacterial toxicity within the human body [4]. Early detection of biofilm structures formed by microorganisms may significantly contribute to the literature by facilitating the prevention and timely treatment of biofilm-associated diseases [5].

Currently, various methods are employed for biofilm detection and analysis, including microscopic techniques, the roll plate method, mass spectrometry, quartz crystal microbalance (QCM)-based methods, impedance-based methods, Raman spectroscopy, the Congo Red method, the crystal violet method in plates or tubes, as well as polymerase chain reaction (PCR) and nuclease-based assays [12,13,14]. However, many of these techniques require expensive instrumentation, are not suitable for high-throughput analysis, and often provide only semi-quantitative results. These limit their use in research aimed at managing biofilm-related challenges [15]. Among the methods cited in the literature, the most commonly used are the crystal violet and Congo Red assays [16].

Due to their cost-effectiveness and ease of execution, these techniques are widely preferred as practical methods in most laboratories. However, some unexpected or inconsistent results have been reported in the literature, as indicated below:

Crystal violet is suitable for liquid cultures and is typically used in tubes or microtiter plates, where it nonspecifically binds to tubes/wells [17]. Although efforts are made to minimize this effect through blank readings, it is sometimes so significant that it becomes difficult to eliminate [17]. Therefore, there is no consensus in the literature regarding the determination of the actual biofilm amount; different formulas are used for calculations, and a standardized method cannot be applied [18,19].

Moreover, Congo Red has been reported to show black color formation in some strains of the same species (e.g., urine, blood) obtained from clinical samples, indicating biofilm formation, while other strains either do not change color or remain as pink colonies, being reported as biofilm-negative [20,21]. It is stated in the literature that Congo Red undergoes spectral shifts when bound to polysaccharides and amyloid-like β-sheet structures, resulting in a darker color [22]. Therefore, is it possible to conclusively state that colorless or pink colonies in these samples do not produce biofilm? To confirm this, these samples should ideally be tested with another biofilm detection method, such as crystal violet, for validation [23]. However, this is often not conducted due to workload constraints or an overreliance on the Congo Red method [20,21]. In another clinical study, when tested, for example, the biofilm-forming abilities of Candida species were defined as strong by crystal violet in 20% of the strains, whereas the Congo Red method identified only 4% [24]. Meanwhile, 35% were classified as moderate by crystal violet, compared to 5% and 37% classified as weak/non-biofilm formers, but Congo Red identified 82% of the strains as biofilm positive [24]. Is this inconsistency due to random error, or does it stem from differences in clinical isolates?

Another issue in biofilm studies is the lack of consensus on which sugar source should be used. While there are publications indicating the use of sucrose and glucose as sugar sources, no standard consensus has been established on this matter [25,26]. Does this issue arise as a result of the different biofilm detection mechanisms of the crystal violet and Congo Red methods, leading to the observation of different results?

To answer these questions and provide scientific insights that may assist researchers working on biofilm studies, this study utilized clinically significant standard bacterial strains (ATCC or NCTC) to conduct biofilm experiments with both methods, and the results were compared. To demonstrate the precise effect of different sugar sources on biofilm formation, LB medium was used to ensure the provision of well-defined culture conditions. Additionally, besides the qualitative comparison of biofilm positive and negative outcomes for both methods, the onset of black color transition in the Congo Red broth method was monitored in real-time and quantified. This also determined how different sugar sources affected the timing of black color transition.

## 2. Materials and Methods

### 2.1. Bacterial Strains

The bacterial strains used in our study that are clinically significant and their order of use in biofilm activity are given in Table 1.

### 2.2. Biofilm Assay

#### 2.2.1. The Congo Red Broth Method

For biofilm studies, three different Congo Red Broth media containing 0.08% Congo Red and different sugar sources (2% glucose, 2% sucrose, 2% fructose, or without sugar) in Luria Bertani (LB) Broth were used. A suspension of 10^6^ CFU/mL was prepared from each bacterial strain and transferred to the medium. All cultures were replicated three times and incubated at 37 °C overnight. Following the methods described in the literature [20,21], samples exhibiting black coloration were evaluated as biofilm-positive, whereas those showing decolorization (transformation to pink) or no color change (remaining red) were evaluated as biofilm-negative.

#### 2.2.2. The Crystal Violet (CV) Method

A suspension of 10^6^ CFU/mL was prepared from each bacterial strain and transferred to Luria Bertani (LB) Broth medium containing 2% glucose, fructose, sucrose, or no sugar. The plates were incubated at 37 °C overnight. Each culture was performed in three replicates. After overnight incubation, analysis was performed using the traditional CV method [19]. For this purpose, the bacterial cultures were removed and the wells were washed with distilled water. A total of 200 µL of 1% CV dye was added to each well and incubated in the dark at room temperature for 15 min. The CV dye was then removed and the wells were washed with water. After drying the plate in a 37 °C incubator for 30 min, 33% acetic acid was added to the wells to dissolve the dye, and absorbance measurements were performed at 590 nm using a Varioscan (Varioscan Lux,. Thermo Fisher. Inc, Waltham, MA, USA) [19]. The evaluation of biofilm-positive and biofilm-negative wells was conducted using the formula employed in the literature for crystal violet quantification [19]. Briefly, wells were considered biofilm-positive if their absorbance values exceeded the mean absorbance of the blank wells (wells without bacteria), calculated from three replicates, plus three times the standard deviation (Mean_blank_ + 3× Standard Deviation). Wells with absorbance values below this threshold were considered biofilm negative.

## 3. Results

### 3.1. Biofilm Assay

**Figure 1:**
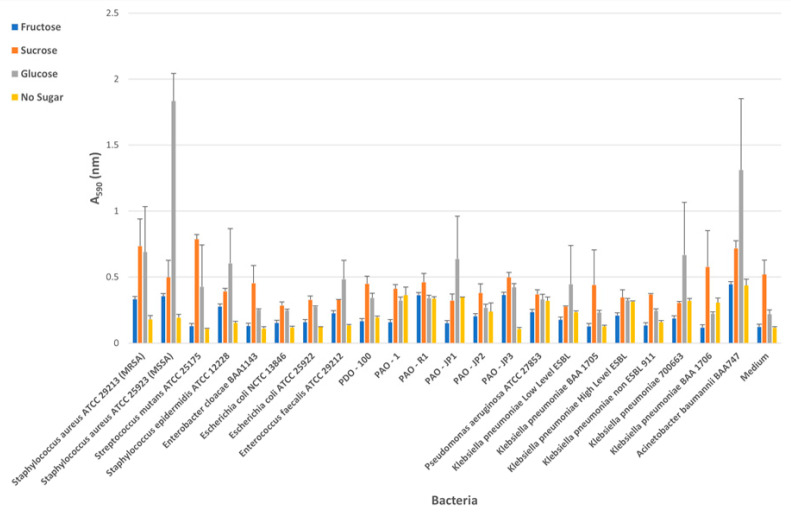
Quantification of biofilm formation of bacterial strains using the crystal violet microtiter plate assay (Error bars represent standard deviation (*n* = 3)).

The results of the biofilm test performed with Congo Red were observed visually. If the color of the plate well was black, it was considered positive; if the color of the well was red, smooth or pink-red, it was considered negative. The absorbance measurement results at 590 nm of the biofilms formed by clinically significant bacterial strains on different sugar sources using the crystal violet method are shown in Figure 1.

**Figure 2:**
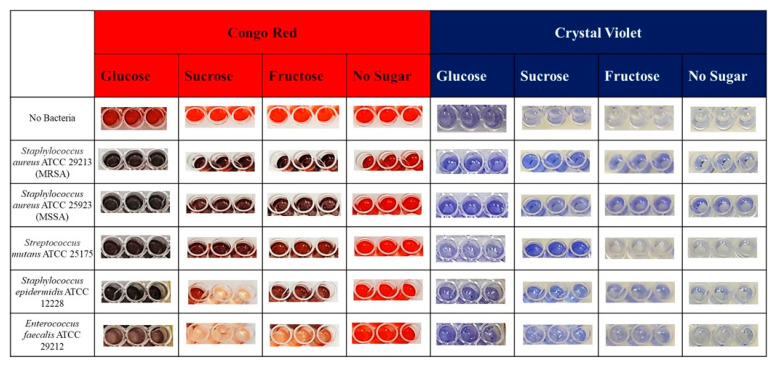
Implant-associated biofilm-forming model bacteria.

When the results of the crystal violet and Congo Red biofilm studies using different sugar sources were evaluated (Figure 2), the implant-associated, biofilm-related model organisms [27] *S. aureus* ATCC 29213 and *S. aureus* ATCC 25923 formed biofilms in both crystal violet and Congo Red assays with all sugar sources, whereas they formed biofilms in sugar-free crystal violet but did not form biofilms in sugar-free Congo Red assays.

*S. mutans* ATCC 25175 formed biofilms in both crystal violet and Congo Red media containing glucose and sucrose (Figure 2). It did not form biofilms in crystal violet medium containing fructose, but biofilm formation was observed in Congo Red medium containing fructose. It was observed that it did not form biofilms in either medium lacking a sugar source.

*S. epidermidis* ATCC 12228 formed biofilms in both crystal violet and Congo Red media containing glucose and fructose (Figure 2). It did not form biofilms in Congo Red medium containing sucrose but did form biofilms in crystal violet medium containing sucrose. It was observed that it formed a biofilm in crystal violet medium without sugar but did not form a biofilm in Congo Red medium without sugar.

*E. faecalis* ATCC 29212 formed a biofilm in crystal violet media containing all sugar sources and glucose-containing Congo red medium but did not form a biofilm in fructose and sucrose-containing Congo red medium (Figure 2). It formed a biofilm in sugar-free crystal violet medium, but did not form a biofilm in sugar-free Congo Red medium.

**Figure 3:**
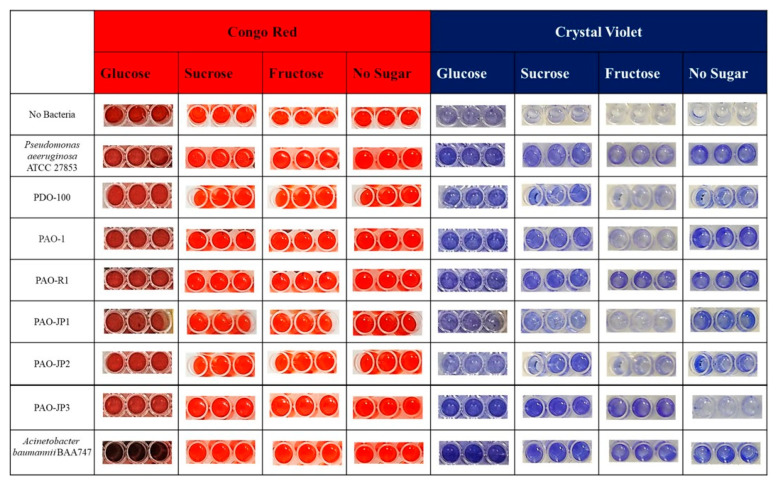
Representative biofilm-forming model strains.

When evaluating the results of crystal violet and Congo Red biofilm studies conducted using different sugar sources, representative biofilm-forming model strains [28] (Figure 3), including *P. aeruginosa* strains (ATCC 27853, PDO 100, PAO-1, PAO-R1, PAO-JP1, PAO-JP2, and PAO-JP3), formed biofilms in crystal violet media containing glucose and sucrose as sugar sources but did not form biofilms in Congo Red.

*P. aeruginosa* ATCC 27853, PAO-R1, and PAO-JP3 were observed to form biofilms in crystal violet media containing fructose but did not form biofilms in Congo Red media containing fructose (Figure 3). PDO-100, PAO-JP1, and PAO-JP2 were found not to form biofilms in either crystal violet or Congo Red media when fructose was present.

*P. aeruginosa* ATCC 27853, PDO 100, PAO-1, PAO-R1, PAO-JP1, and PAO-JP2 were observed to form biofilms in crystal violet media without a sugar source but did not form biofilms in Congo Red (Figure 3). PAO-JP3, however, was found not to form biofilms in either crystal violet or Congo Red media without a sugar source.

*A. baumannii* BAA-747 was observed to form biofilms in both glucose-containing crystal violet and Congo Red media (Figure 3). It formed biofilms in sucrose- and fructose-containing crystal violet media but did not form biofilms in Congo Red media. It was observed that it formed a biofilm in sugar-free crystal violet medium but did not form a biofilm in sugar-free Congo Red medium.

**Figure 4:**
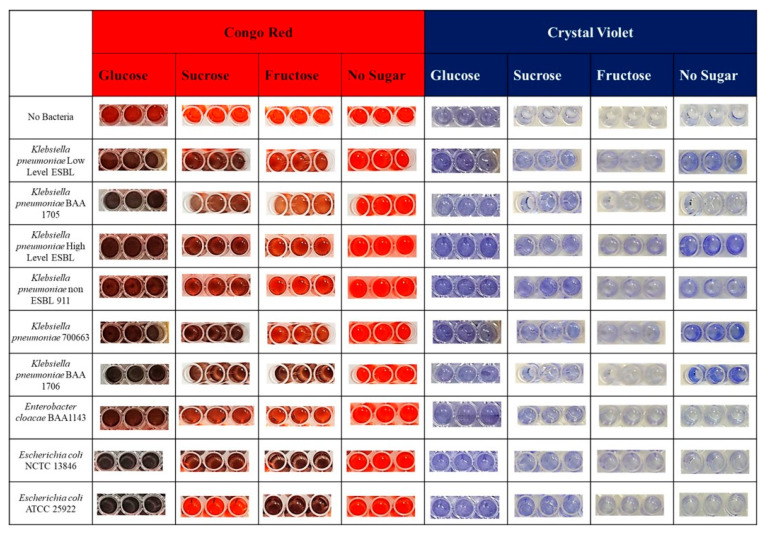
Multi-drug resistant, biofilm-forming model bacteria.

When evaluating the results of the crystal violet and Congo Red biofilm studies conducted using different sugar sources, multi-drug resistant, biofilm-resistant model organisms [29] (Figure 4), including *K. pneumoniae* Low Level ESBL, *K. pneumoniae* BAA 1705, *K. pneumoniae* High Level ESBL, *K. pneumoniae* non-ESBL 911, *K. pneumoniae* ATCC 700663, and *K. pneumoniae* BAA 1706, were observed to form biofilms in crystal violet and Congo Red media containing glucose and sucrose.

*K. pneumoniae* High Level ESBL and K. pneumoniae BAA 1706 were observed to form biofilms in fructose-containing crystal violet media but did not form biofilms in Congo Red media (Figure 4). *K. pneumoniae* Low Level ESBL, *K. pneumoniae* BAA 1705, *K. pneumoniae* non-ESBL 911, and *K. pneumoniae* ATCC 700663 did not form biofilms in either crystal violet or Congo Red media containing fructose.

*K. pneumoniae* Low Level ESBL, High Level ESBL, non-ESBL 911, *K. pneumoniae* ATCC 700663, and BAA 1706 were observed to form biofilms in crystal violet media without a sugar source but did not form biofilms in Congo Red (Figure 4). *K. pneumoniae* BAA 1705 was found not to form biofilms in either crystal violet or Congo Red media without a sugar source.

*E. coli* NCTC 13846 has been observed to form biofilms in both crystal violet and Congo Red media containing glucose and sucrose (Figure 4). It was observed that it did not form biofilms in crystal violet medium containing fructose but did form biofilms in Congo Red medium containing fructose. It was observed that it did not form biofilms in either crystal violet or Congo Red media without a sugar source.

*E. coli* ATCC 25922 was observed to form biofilms in both crystal violet and Congo Red media containing glucose (Figure 4). While biofilms were observed in crystal violet media containing sucrose, no biofilms were observed in Congo Red. It was observed that it did not form a biofilm in crystal violet media containing fructose, but did form a biofilm in Congo Red. It was observed that it formed a biofilm in crystal violet medium without a sugar source, but it did not form a biofilm in Congo Red medium without sugar.

*E. cloacae* BAA1143 was observed to form biofilms in crystal violet media containing glucose and in Congo Red media (Figure 4). It was observed to form biofilms in crystal violet media containing sucrose, but not in Congo Red. It was observed that it did not form biofilms in either crystal violet or Congo Red media when fructose or other sugar sources were not present.

### 3.2. Comparison of Biofilm Assays

When the data obtained from biofilm formation of each bacterial strain using both methods and different sugar sources were compiled into a comparative table of biofilm-positive and biofilm-negative results, the consistency between these two methods in determining the presence of biofilm is shown in Table 2.

Accordingly, for *S. aureus* ATCC 29213, *S. aureus* ATCC 25923, *S. epidermidis* ATCC 12228, *E. faecalis* ATCC 29212, *E. coli* ATCC 25922, *P. aeruginosa* strains (PDO-100, PAO-1, PAO-R1, PAO-JP1, PAO-JP2 and ATCC 27853), *K. pneumoniae* Low Level ESBL, *K. pneumoniae* High Level ESBL, *K. pneumoniae* non-ESBL 911, *K. pneumoniae* ATCC 700663, *K. pneumoniae* BAA 1706, and *A. baumannii* BAA747, the results of biofilm assays without sugar were inconsistent between the two methods. Specifically, biofilms tested positive by the crystal violet method but negative by the Congo Red method. Since these represent 17 of the 22 bacterial strains tested, the inconsistency between the two methods without sugar corresponded to approximately 77% (Table 2).

Biofilm assays conducted in the presence of sucrose for *E. faecalis* ATCC 29212, *E. cloacae* BAA1143, *E. coli* ATCC 25922, *P. aeruginosa* strains (PDO-100, PAO-1, PAO-R1, PAO-JP1, PAO-JP2, PAO-JP3 and ATCC 27853) and *A. baumannii* BAA747 were observed to be positive using the crystal violet method but negative using the Congo Red method. Since these represent 11 out of the 22 bacterial strains tested, the inconsistency between the two methods in the presence of sucrose corresponds to approximately 50% (Table 2).

In biofilm formation assays using glucose, inconsistency between the two methods was observed only with *P. aeruginosa* strains (PDO-100, PAO-1, PAO-R1, PAO-JP1, PAO-JP2, PAO-JP3, and ATCC 27853), where biofilm-negative results obtained by the Congo Red method corresponded to biofilm-positive results by the crystal violet method. Since these represent 7 out of the 22 bacterial strains tested, the inconsistency between the two methods in the presence of glucose was approximately 32% (Table 2).

In biofilm assays containing fructose, the Congo Red method yielded biofilm-negative results for *P. aeruginosa* strains (PAO-R1, PAO-JP3 and ATCC 27853), *E. faecalis* ATCC 29212, *K. pneumoniae* High Level ESBL, *K. pneumoniae* BAA 1706, and *A. baumannii* BAA747, while the crystal violet method showed biofilm-positive results for these strains. Conversely, for *S. mutans* ATCC 25175, *E. coli* NCTC 13846, and *E. coli* ATCC 25922, the opposite was observed: biofilm-negative results were obtained with the crystal violet method, whereas the Congo Red method yielded biofilm-positive results. Since these represent 10 out of the 22 bacterial strains tested, the inconsistency between the two methods in the presence of fructose was approximately 45% (Table 2).

## 4. Discussion

The World Health Organization has identified biofilm-associated multidrug-resistant bacteria as a serious global health and environmental threat [30]. Biofilms are structured microbial communities embedded in an extracellular polymeric substance (EPS) that adhere to both living and non-living surfaces, progressing through stages of adhesion, colonization, maturation, and dispersion [31]. Due to their resistance to immune responses and high antibiotic concentrations, biofilms can cause persistent inflammation and are difficult to eradicate [31]. They are known to impair immune cell activity, particularly by inhibiting leukocyte movement and reducing reactive oxygen species (ROS) production, which are crucial for bacterial elimination [32]. Clinically relevant pathogens such as *E. coli*, *K. pneumoniae*, *P. aeruginosa*, *S. aureus*, *S. epidermidis*, *E. cloacae*, *E. faecalis*, *S. mutans*, *A. baumannii* are capable of forming biofilms, especially on medical devices like catheters, prosthetics, and heart valves, leading to long-term infections and tissue damage [7]. Early detection of biofilm formation is therefore essential for effective prevention and timely intervention. While various techniques such as microscopy, PCR, mass spectrometry, Raman spectroscopy, and staining assays like crystal violet and Congo Red are used to study biofilms, many are limited by high costs, low throughput, and semi-quantitative results [12,13,14,15,16].

Certain pathogenic microorganisms such as *E. coli*, *K. pneumoniae*, *P. aeruginosa*, *S. aureus*, *S. epidermidis*, *E. cloacae*, *E. faecalis*, *S. mutans*, and *A. baumannii* are well-known for their ability to form biofilms, particularly in clinical settings [7,30]. Among these, species like *S. aureus*, *S. epidermidis*, *E. faecalis*, and *S. mutans* are known to cause significant biofilm-related infections on implanted medical devices, contributing to severe clinical consequences [30,33,34,35]. On the other hand, organisms such as *P. aeruginosa*, *A. baumannii*, *E. coli*, *E. cloacae*, and *K. pneumoniae* are of particular concern due to their ability to develop high levels of multidrug resistance through biofilm formation [36,37,38]. Biofilms not only complicate treatment processes but also reduce patient quality of life and contribute to substantial economic burdens on healthcare systems [30]. As a result, research on biofilm-related mechanisms has gained momentum, with a growing interest in understanding the genetic and molecular basis of biofilm development [39]. To facilitate these studies, model bacterial strains such as PDO-100, PAO-1, PAO-R1, PAO-JP1, PAO-JP2, and PAO-JP3 have been developed, which enable researchers to investigate the effects of specific genetic modifications on biofilm formation [40].

In the crystal violet assay, the exopolysaccharide matrix produced by biofilm-forming bacteria is stained, allowing researchers to distinguish between biofilm-producing and non-producing cultures based on the intensity of staining [41]. However, since crystal violet is also a component of the Gram staining method and can bind to bacterial cell walls, it also has the potential to stain bacteria within the biofilm [41]. Additionally, it may nonspecifically adhere to the surfaces of tubes or plate wells used for culturing [17]. To address this issue, background staining (non-specific binding) is typically subtracted from the total absorbance values using various correction formulas, in order to determine whether a sample is biofilm-positive or negative [18,19]. Nevertheless, because the degree of non-specific staining can vary considerably, this introduces uncertainty and variability in the results [17].

Another commonly used method, the Congo Red assay, involves culturing bacteria on a red-colored medium [38]. In this method, biofilm-positive strains are characterized by a visible color change from red to black [20,21,41]. In contrast, cultures that show no color change or become decolorized (turning pink) are considered biofilm-negative [20,21,41].

Studies based on clinical samples have shown that different strains of the same bacterial species, even when isolated from the same clinical specimen, can exhibit varying capacities for biofilm formation [20,21,22,23,24]. While some strains may test positive, others may yield negative results [20,21,22,23,24]. Although these differences can be attributed to strain-level genetic variability, the interpretation of biofilm-positive or -negative outcomes is often limited by the reliability of the detection method employed, as most studies rely on a single assay. Interestingly, some reports have demonstrated discrepancies when both the crystal violet and Congo Red methods are used in parallel, with inconsistent results between the two assays [20,21,22,23,24,42]. This disagreement likely stems from fundamental differences in their detection mechanisms such as black pigmentation, pink discoloration, or violet staining as well as the limitations inherent in each method. For instance, the underlying mechanism of Congo Red-based detection remains poorly understood, while the nonspecific staining behavior of crystal violet is highly variable and may interfere with accurate interpretation. Moreover, due to the incomplete understanding of these mechanisms, the role of carbon sources such as specific sugars used during culture in biofilm formation is also not fully established. There is no clear consensus in the literature regarding a standardized sugar source for biofilm induction; however, glucose and sucrose are most commonly used [25,26,42]. Although various studies have explored the impact of different sugars on biofilm development, to the best of our knowledge, no comprehensive comparative study has been conducted to assess whether different sugar sources influence the performance or reliability of the crystal violet and Congo Red methods, using standard bacterial strains in a comparative manner.

Given the inconsistencies observed between the crystal violet and Congo Red assays, there is a growing need for clearer comparative analysis of these two widely used biofilm detection methods. In this study, biofilm cultures were prepared using clinically relevant bacterial strains, including commonly used model strains such as ATCC and NTCC strains. Both the crystal violet and Congo Red methods were applied to these cultures to enable a direct comparison of their outcomes. Additionally, the study investigated the impact of different sugar sources, an important yet often overlooked parameter, on the performance of each method. Numerous studies in the literature have investigated sugar use in in vitro experiments with bacteria that cause pathogenic effects in humans, such as *E. coli*, *P. aeruginosa*, *K. pneumoniae*, *S. aureus*, and *S. mutans*. The results of these studies suggest that culture media containing added sugar inhibit bacterial growth or cause stress to the bacteria [43,44]. Biofilm cultures were designed using various sugar conditions, including glucose, sucrose, fructose, and sugar-free media, in order to assess how these carbon sources, influence biofilm detection results across both assays.

The results revealed significant discrepancies between the two methods, strongly related to sugar content. Of the 22 strains tested, 17 (77%) showed opposite biofilm results in sugar-free media: positive by crystal violet but negative by Congo Red. Similar inconsistencies appeared with glucose (32% of strains) and sucrose (50% of strains). Fructose also caused discrepancies but with a more complex pattern: some strains (PAO-R1, PAO-JP3, *P. aeruginosa* ATCC 27853, *E. faecalis* ATCC 29212, *K. pneumoniae* High-Level ESBL, *K. pneumoniae* BAA 1706, *A. baumannii* BAA 747) tested negative by Congo Red but positive by crystal violet, whereas others (*S. mutans* ATCC 25175, *E. coli* NCTC 13846, *E. coli* ATCC 25922) showed the reverse. Overall, fructose caused inconsistencies in approximately 45% of strains.

The bacteria defined as biofilm producers in the literature and used in this study (*S. aureus*, *S. mutans*, *E. coli*, *E. faecalis*, *K. pneumoniae*, *P. aeruginosa* and *A. baumannii*) were found to be positive by the crystal violet method, consistent with the literature, while showing weak or negative biofilm activity with Congo Red. These inconsistencies can be explained by several factors. First, genetic variation at the strain level can lead to significant differences in biofilm regulatory genes and pathways even within the same species. Second, environmental conditions such as nutrient composition, glucose concentration, pH, temperature, and oxygen availability are known to affect biofilm development. Third, there are methodological differences between studies. These differences highlight the necessity of environmental optimization and method integration when evaluating biofilm formation [41,42,43,44,45].

The inconsistencies observed between the two methods align with the varying numbers of biofilm-positive and -negative results reported in the literature [20,21,22,23,24,42], especially when the crystal violet and Congo Red assays are applied to the same clinical samples. These findings clearly indicate that the two methods operate through different mechanisms, and a biofilm-negative result in one method does not necessarily imply a true negative, and vice versa. Another important observation is that the sugar content in the culture medium significantly influences the detection mechanism of these assays, or potentially affects biofilm formation itself, although this is not yet fully understood. Notably, the highest agreement between the two methods (approximately 68%) was observed when glucose was used as the carbon source.

## 5. Conclusions

In conclusion, to ensure accurate reporting of biofilm formation in tested samples, it is essential to optimize the choice of sugar sources according to the bacterial strain used and, importantly, to apply both methods concurrently to achieve a consensus biofilm-positive or biofilm-negative result. These findings provide valuable insights into the mechanisms of biofilm formation and serve as a guide for researchers. Rapid completion of such studies is urgently needed to deepen our understanding of biofilm mechanisms and to develop reliable diagnostic methods.

## Figures and Tables

**Table 1:** Bacterial strains.

Bacterial Strains	Gram Staining Morphology
*Staphylococcus aureus* ATCC 29213 (MRSA)	Gram (+)
*Staphylococcus aureus* ATCC 25923 (MSSA)	Gram (+)
*Streptococcus mutans* ATCC 25175	Gram (+)
*Staphylococcus epidermidis* ATCC 12228	Gram (+)
*Enterobacter cloacae* BAA1143	Gram (−)
*Enterococcus faecalis* ATCC 29212	Gram (+)
*Escherichia coli* NCTC 13846	Gram (−)
*Escherichia coli* ATCC 25922	Gram (−)
*Pseudomonas aeruginosa* (PDO—100)	Gram (−)
*Pseudomonas aeruginosa* (PAO—1)	Gram (−)
*Pseudomonas aeruginosa* (PAO—R1)	Gram (−)
*Pseudomonas aeruginosa* (PAO—JP1)	Gram (−)
*Pseudomonas aeruginosa* (PAO—JP2)	Gram (−)
*Pseudomonas aeruginosa* (PAO—JP3)	Gram (−)
*Pseudomonas. aeruginosa* ATCC 27853	Gram (−)
*Klebsiella pneumoniae* Low Level ESBL	Gram (−)
*Klebsiella pneumoniae* BAA 1705	Gram (−)
*Klebsiella pneumoniae* High Level ESBL	Gram (−)
*Klebsiella pneumoniae* non ESBL 911	Gram (−)
*Klebsiella pneumoniae* 700663	Gram (−)
*Klebsiella pneumoniae* BAA 1706	Gram (−)
*Acinetobacter baumannii* BAA747	Gram (−)

**Table 2:** Comparison of The Congo Red Broth Method and The Crystal Violet Methods.

Bacteria	CV Glucose	CR Glucose	CV Fructose	CR Fructose	CV Sucrose	CR Sucrose	CV No Sugar	CR No Sugar
*S. aureus* ATCC 29213	+	+	+	+	+	+	+	−
*S. aureus* ATCC 25923	+	+	+	+	+	+	+	−
*S. mutans* ATCC 25175	+	+	−	+	+	+	−	−
*S. epidermidis* ATCC 12228	+	+	+	+	+	−	+	−
*E. cloacae* BAA1143	+	+	−	−	+	−	−	−
*E. faecalis* ATCC 29212	+	+	+	−	+	−	+	−
*E. coli* NCTC 13846	+	+	−	+	+	+	−	−
*E. coli* ATCC 25922	+	+	−	+	+	−	+	−
*P. aeruginosa* (PDO—100)	+	−	−	−	+	−	+	−
*P. aeruginosa* (PAO—1)	+	−	−	−	+	−	+	−
*P. aeruginosa* (PAO—R1)	+	−	+	−	+	−	+	−
*P. aeruginosa* (PAO—JP1)	+	−	−	−	+	−	+	−
*P. aeruginosa* (PAO—JP2)	+	−	−	−	+	−	+	−
*P. aeruginosa* (PAO—JP3)	+	−	+	−	+	−	−	−
*P. aeruginosa* ATCC 27853	+	−	+	−	+	−	+	−
*K. pneumoniae* Low Level ESBL	+	+	−	−	+	+	+	−
*K. pneumoniae* BAA 1705	+	+	−	−	+	+	−	−
*K. pneumoniae* High Level ESBL	+	+	+	−	+	+	+	−
*K. pneumoniae* non ESBL 911	+	+	−	−	+	+	+	−
*K. pneumoniae* ATCC 700663	+	+	−	−	+	+	+	−
*K. pneumoniae* BAA 1706	+	+	+	−	+	+	+	−
*A. baumannii* BAA747	+	+	+	−	+	−	+	−

## Data Availability

The original contribution presented in this study are included in the article. Further inquiries can be directed to the corresponding author.
